# Simultaneous two organ metastases of the giant basal cell carcinoma of the skin

**DOI:** 10.1186/1477-7800-2-1

**Published:** 2005-01-04

**Authors:** Eray Copcu, Alper Aktas

**Affiliations:** 1 Plastic and Reconstructive Surgery Department, Medical Faculty, Adnan Menderes University, Aydin, Turkey; 2 Plastic and Reconstructive Surgery Department, Samsun State Hospital, Samsun, Turkey

## Abstract

**Background:**

Basal Cell Carcinoma (BCC) is the most common carcinoma in humans. It accounts for 20% of carcinomas in men and 10–15% of carcinomas in women. Despite its high incidence, metastatic events are exceedingly rare. The reported frequency of metastatic dissemination is estimated at 0.0028–0.5 percent. Once metastasis is detected, there is a high mortality rate of 50% within 8 months.

**Methods:**

In this study, we present a case of simultaneous lung and parotid metastases of giant BCC primary located on the right medial canthus of a 62 year old female.

**Results:**

Examination of the tumor located on the medial canthus obtained showed "adenoid BCC". Computed tomography (CT) was performed to evaluate parotid region for evaluation of parotid gland and chest. Parotid and lung metastasis were detected in CT. Routine labarotory tests and radiological investigations were done. There was no abnormal finding. We also investigated this patient with a bone scan (normal), abdominal and cranial CT scans (also normal).

**Conclusion:**

Although metastasis of BCC is a very rare condition, this study reports a case of simultaneous parotid gland and lung metastasis originating from a giant BCC primary that was located on the right inner canthus of a 62 year old female.

## Background

Basal cell carcinoma (BCC) is the most common carcinoma in humans and accounts for 20% of carcinomas in men and 10–15% of carcinomas in women. Approximately 75–86% of primary BCCs are found on the head or neck. The most common location on the head is the nose, specifically the nasal tip and alae. It constitutes 90% of periorbital malignancies [1,2]. Sun exposure is the primary etiologic agent for the development of BCC. The tumors are more frequent in individuals with fair complexions.

BCC arising on the medial canthus tends to be deep and invasive and may result in perineural extension and loss of optic nerve function. Pieh et al reported that the highest recurrence rates of BCC following attempted excision, (approximately 60%), was seen with lesions arising from the medial canthus since these lesions tend to be more invasive and difficult to manage [3]. Reclusive patients or patients who neglect the lesions for long periods of time are more likely to have giant, invasive tumors [4]. Giant BCC, defined as lesions more than 5 cm at its largest diameter, are rare forms of BCC [4]. Giant BCCs more commonly appear on the trunk and display a more aggressive behavior, resulting in local invasion and metastasis. The reported incidence of metastatic BCC ranges from 0.03 % to 0.55 [5]. We report a case of simultaneous lung and parotid gland metastases of giant BCC located on medial canthus.

## Case report

A 62-year-old woman was referred to the Plastic and Reconstructive Surgery Department for treatment of a bleeding exophytic tumor located on the right inner canthus. She had had the lesion for approximately 11 years. Initially, the patient was treated with excision and primary closure ten years ago. At this time the tumor had a diameter of 5 cm. The tumor was diagnosed as adenoid BCC microscopically and surgical margins were tumor-positive. The patient was operated on two years later when the diameter of the recurrent tumor was 15 mm. Histological examination of this second specimen revealed an "adenoid BCC" with clear surgical margins.

**Figure 1:**
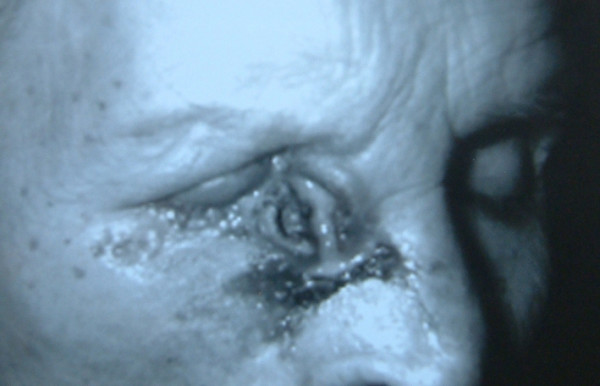
Giant BCC located on the inner canthus

**Figure 2:**
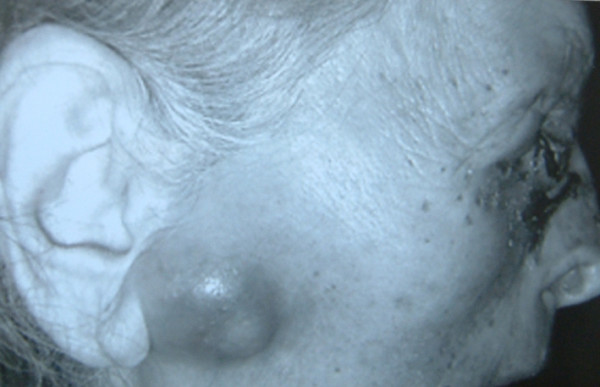
Involvement of the parotid gland of the patient

Although the tumor recurred again after the second excision, the patient neglected medical advice and did not undertake any treatment (Figure 1). More recently, however, the tumor began growing rapidly and became hemorrhagic. On examination the lesion was located on the right inner canthus and involved 1/3 of the eyelid. The size of tumor was approximately 55 mm × 45 mm. Visual functions of the patient were normal. However, a fixed mass developed in the patient's periauricular area six months ago (Figure 2), although there were no palpable cervical nodes. We therefore investigated this region with computed tomography (CT), which revealed a tumor involving the right orbital structures extending to the ethmoidal cells. The tumor also involved the right parotid gland and multiple cervical lymph nodes.

**Figure 3:**
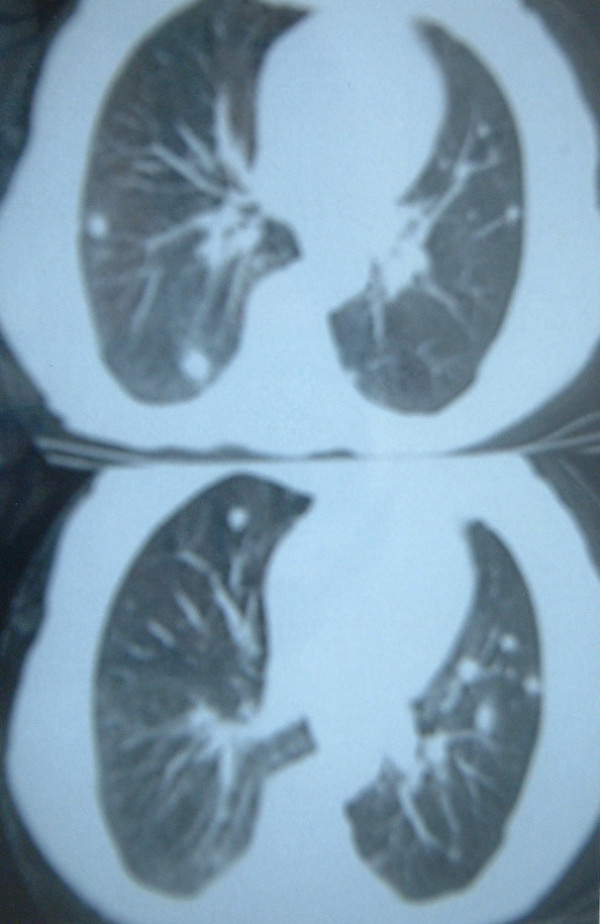
CT scan of the chest of the patient. Multiple metastatic lesions were seen.

We also investigated this patient with a bone scan (normal), abdominal and cranial CT scans (also normal) and a thoracic CT. Multiple metastatic lesions were seen in the chest CT (Figure 3).

**Figure 4:**
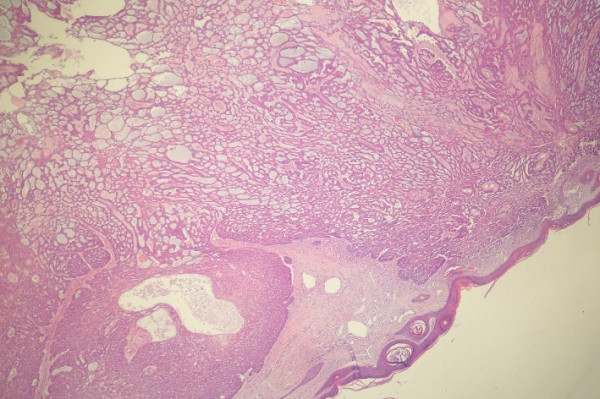
Histological view of the specimen showed "adenoid BCC". (Hematoxylene eosin staining, × 50 magnification)

Examinations of the cardiovascular, gastrointestinal, neurological, urogenital and hematological systems and other parts of the skin were performed by physical and routine laboratory and radiological techniques. There were no abnormal findings. Biopsy was performed from the tumor located on the inner canthus and revealed "adenoid basal cell carcinoma" (Figure 4). Also, Fine needle biopsies were performed on the parotid and pulmonary lesions which confirmed the presence of adenoid BCC in these regions.

The patient did not accept the offer of surgical treatment for the tumor on the inner canthus. She was referred to the Oncology Department and treated with radiotherapy and chemotherapy. The patient received approximately 6000 cGy of external beam radiation over 3 weeks totally. Also, chemotherapy was initiated with cisplatin and 5-fluoruracil. She was followed up with physical examination and CT scans for six months and there were no metastases to other organs. She is still being followed.

## Discussion

Spates et al. noted that metastatic BCC was first reported in 1894 [6]. As outlined by Lattes and Kessler, metastatic basal cell carcinoma is defined by the following criteria:

1) the primary tumor must occur in skin containing hair follicles and not the mucous membranes;

2) metastasis cannot be by simple extension, but occurring at a site distant from the primary tumor;

3) the primary tumor and the metastasis must have similar histologic appearances of basal cell carcinoma; and

4) squamous cell features must not be present in the lesions [7,8]. The case presented here satisfied these three criteria. More than 300 cases of metastatic BCC have been reported in the literature. Two-thirds of metastatic BCCs arise from primary tumors on the face, with the ear being the most common location. Higher rates of metastasis also occur from primary lesions on the scalp and genitalia [9-11]. Primary BCC metastasizes through hematogenous and lymphatic routes. As was the case with our patient, metastasis to the lymph nodes has been estimated to occur in 70% of cases. The most common organs involved in hematogenous spread are lungs, bone, and skin [12,13].

While the usual BCC that gives rise to metastases is a large, ulcerated, locally invasive BCC of the head and neck that recurs despite repeated surgical procedures or radiotherapy, these features are not absolute prerequisites for metastasis [14]. Immunosupression may be a factor in the pathogenesis of the metastasis of BCC, but there was no finding suggestive of immune system abnormality in the case presented here [14]. Some authors have suggested that immunosuppression or impaired cell-mediated immunity (including AIDS) may predispose to BCC and BCC metastasis [15-17]. BCC usually has multiple skin recurrences before metastases become evident as in the case presented here [18]. BCCs with any of the following: long duration, location in the mid face or ear, a diameter larger than 2 cm, with aggressive histological subtype, previous treatment, neglected, or a history of radiation exposure, should be considered "high risk [19]. Although giant BCC is commonly located on the trunk, Takemato et al reported a case of with giant basal cell carcinoma which invaded the orbital tissue and anterior skull base [4]. They concluded that giant basal cell carcinomas have aggressive character to destroy tissue and more metastatic potential. Many investigators have reported that radical treatment with a wide excision of the tumor at an early stage is essential to treat a potentially aggressive BCC. Takemato et al used a free rectus abdominis musculocutaneous flap in the treatment of giant BCC which invaded the orbital tissue [4].

Tumors greater than 3 cm in diameter have a 2 % incidence of metastasis and/or death. This increases to 25% in those lesions more than 5 cm in diameter and to 50% in lesions more than 10 cm in diameter [20]. The prognosis of metastatic BCC is extremely poor. Once metastasis is detected, there is a high mortality rate of 50% within 8 months [20]. This poor outcome has led to the use of systemic chemotherapy in a number of individual cases. Several chemotherapeutic agents that have been used in metastatic BCC, including fluorouracil and combination of vincristine, bleomycine and prednisone [18].Kaufman suggested that cisplatin, alone or in combination is probably the most active in metastatic BCC [21]. Recently, Jefford et al presented their experience in the treatment of metastatic BCC [22]. According to their study, systemic chemotherapy with cisplatin and paclitaxel provided palliative benefit to their patient with acceptable toxicity and conclude that the regimen is a reasonable choice for the rare patient presenting with metastatic BCC. About half of metastatic BCCs have metastasis to lymph nodes as the first site, but hematogenous route to lung and bone is also equally represented [23]. Robinson and Dahiya reported a case of BCC with pulmonary and lymph node metastasis causing death [14]. BCC located on the nose, eyebrow, ear, nose, and temple frequently metastasizes to the parotid and lymph nodes of neck [24]. To the best of our knowledge this report is the first to present simultaneous lung and parotid gland metastases of giant BCC.

## Competing interests

The author(s) declare that they have no competing interests.

## Authors' contributions

EC, conceived the study and coordinated the write-up and submission. AA participated in the writing of the manuscript. All authors read and approved the final manuscript.
